# Illness, Self-Rated Health and Access to Medical Care among Waste Pickers in Landfill Sites in Johannesburg, South Africa

**DOI:** 10.3390/ijerph17072252

**Published:** 2020-03-27

**Authors:** Felix Made, Vusi Ntlebi, Tahira Kootbodien, Kerry Wilson, Nonhlanhla Tlotleng, Angela Mathee, Mpume Ndaba, Spo Kgalamono, Nisha Naicker

**Affiliations:** 1 Epidemiology and Surveillance Section, National Institute for Occupational Health, National Health Laboratory Service, Johannesburg 2000, South Africa; VusiNT@nioh.ac.za (V.N.); TahiraK@nioh.ac.za (T.K.); KerryW@nioh.ac.za (K.W.); NonhlanhlaT@nioh.ac.za (N.T.); NishaN@nioh.ac.za (N.N.); 2 Faculty of Health Sciences, School of Public Health, University of Witwatersrand, Johannesburg 2000, South AfricaSpoK@nioh.ac.za (S.K.); 3 Department of Environmental Heath, Faculty of Health Sciences, University of Johannesburg, Johannesburg 2000, South Africa; 4 Environment and Health Research Unit, South African Medical Research Council, Johannesburg 2000, South Africa; 5 Occupational Medicine Section, National Institute for Occupational Health, National Health Laboratory Service, Johannesburg 2000, South Africa; NompumeleloN@nioh.ac.za

**Keywords:** mental illness, infectious diseases, chronic diseases, clinic visits, self-rated health, waste pickers

## Abstract

Waste pickers are exposed to various environmental health hazards, and self-rated health (SRH) could influence their medical care access. This study investigated the association between illness, clinic visits and SRH, and assessed if SRH can increase clinic visits. A cross-sectional study was conducted. SRH was defined as “very good”, “good”, “fair”, and “poor”. The illnesses were mental health, infectious, and chronic diseases. Medical care access included clinic visits in the previous 12 months. An ordinal logistic regression model was fitted to assess the association. There were 361 participants, 265 (73.41%) were males. Median age was 31 years, (interquartile range (IQR): 27–39). SRH: poor (29.89%), fair (15.92%), good (43.30%) very good (10.89%). Ever smoked (adjusted odds ratio (AOR): 1.72; 95% confidence interval (CI): 1.11–2.66), mental health (AOR: 1.87; 95% CI: 1.22–2.84), chronic (AOR: 2.34; 95% CI:1.47–3.68) and infectious (AOR: 2.07; 95% CI: 1.77–3.63) diseases were significantly associated with increased odds of reporting poor health. Clinic visit was not associated with SRH. From 99 (31%) individuals who rated their health as poor and ill, 40% visited a clinic (*p* = 0.0606). Acute and chronic illnesses were associated with poor SRH but this did not increase clinic visits. Provision of mobile clinic services at the landfill sites could increase access to medical care.

## 1. Introduction

Informal waste pickers make a living by collecting and selling recyclable materials from the municipal solid waste dumps or landfill sites. Waste pickers are marginalised individuals who pick and sort waste for sale but do so under a high risk of exposure to environmental and occupational health hazards [1]. A high unemployment rate (26.7%) and increased urbanisation, are associated with a rise in the informal economy, such as waste recycling in South Africa (SA) [2]. As a result, informal waste pickers are a common sight on landfill sites and the streets of cities around the world, as millions of people make a living collecting, sorting, recycling and selling valuable material disposed of as waste [3]. There are an estimated 90,000 waste pickers in South Africa, and the number could be much higher due to the rising unemployment rate [4]. In Johannesburg, yearly there are about 1.5 million tonnes of waste generated and the per capita generation of waste is about 0.7 kg per day [5,6]. Most of the waste pickers work at designated places called landfill sites which hold large quantities of waste, while others pick waste from the streets. The South African Department of Environmental Affairs in 2012 reported that at least 90% of the general waste produced ended up at the landfill sites [7].

In most developing countries, including SA, wastes are disposed in landfill sites, which poses environmental risks [8] to nearby communities and more specifically, to the waste pickers themselves. Waste pickers are highly exposed to various hazards as they work through organic waste, including toxic medical waste [9]. Inadequate planning and management of waste disposal such as chemicals, batteries, and medical waste increases the risk of disease and infection among waste pickers [3]. There are undoubtedly inadequate health and safety measures in place, including the use of personal protective equipment (PPE) and awareness of the hazards that have a significant detrimental impact on health. Thus, the health of a large proportion of waste pickers can potentially be adversely affected.

Waste pickers have a higher risk of acquiring a number of diseases and injuries compared to the general population. This is because of exposure to cuts (lesions to the skin) and toxic substances like chemicals, bacteria, dust inhalation, and medical wastes [9,10,11,12]. Diseases they most commonly face include respiratory infections, skin diseases, mental illness, and chronic diseases [11,13]. Social stigma may affect the mental state and emotional wellbeing of the waste pickers [3]. The risk of infection is even higher since it can arise from cuts, needle pricks and ingestion of organic waste found at landfills [14].

Due to the demand for money to feed their families, waste pickers often do not look after their health, potentially putting themselves at risk. Waste pickers who think their health is poor or fair are more likely to die sooner than those who are very satisfied, according to Frankenberg and Jones [15]. Self-rated health (SRH) was found to be a useful tool, similar to other measurements [16,17], which may be a good predictor of morbidity among waste pickers. Several factors including age, education, income, smoking, alcohol and chronic diseases, influence SRH [18]. Seeking medical care by attending clinics may also be an important determinant of health perception. For instance, in Guatemala, it was reported that waste pickers are less likely to visit clinics and hospitals when they fall sick because of fear of stigmatisation and discrimination [19]. Previous studies reported the association of SRH demographic factors, clinical and psychological factors including depression [20,21,22]. Age, sex, education, income, and smoking were all associated with SRH in the general population [23,24,25], but there are limited studies looking at how these factors predict SRH among waste pickers.

Investigating the association between illness, medical care access and SRH among waste pickers, since they work in a potentially health hazardous environment is important in reducing the burden of disease. Illness and poor self-rating of health should influence or improve clinic visits so that morbidity is reduced. Decreased access to medical care could indicate poor health-seeking behaviour among waste pickers. Accordingly, workers’ beliefs and representations determine whether a positive or negative feeling develops about their health and quality of life and medical care access. To our knowledge there is no available information on how SRH increases medical care access utilisation among waste pickers. Understanding how these factors influence SRH can contribute to planning, monitoring and evaluation of interventions aimed at reducing and unpacking help-seeking behaviour to improve the health of waste pickers. The objective of this study was to investigate the association between illness, medical care access and SRH as well as to assess if the participants’ ratings increase clinic visits among waste pickers in Johannesburg, South Africa (SA).

## 2. Materials and Methods

### 2.1. Study Design and Setting

This study was a cross-sectional survey conducted at two of the largest landfill sites in Johannesburg. One of the study sites in the west of Johannesburg had approximately 600 waste pickers and the second one, which is in the south-west of Johannesburg, had approximately 3000 waste pickers. A proportionate number of participants were sampled from these two sites. These landfill sites were chosen because of their proximity to the most densely populated area and they had the largest number of waste pickers.

### 2.2. Study Population and Sample

Male and female waste pickers aged 18 years or older working in both of the landfill sites were included in this study. All waste pickers available on the day of the study were approached and the aim of the study was thoroughly explained to them; those who consented were, therefore, included in the study. Based on the proportion of reported chronic diseases [26], an estimated sample size of 340 was required at the power of 88% to detect two times more likely difference (Odds ratio (OR) 2.02) in reporting poor SRH and clinic visits compared to waste pickers with no history of chronic diseases, good SRH and no clinic visits. The sample size was estimated using two-sided test at the 5% level of statistical significance. During data collection, 373 participants volunteered and were interviewed to cater for missing information. A convenience sampling strategy was used to recruit waste pickers who were available on the day of data collection and consented to the study. A total of 361 with a 97% response rate was included in this analysis, and 12 were removed because of missing information. Ethical clearance for this study was obtained from the Human Research Ethics Committee (HREC) of the University of the Witwatersrand with clearance number: M171120.

### 2.3. Data Collection Tools and Methods

A structured questionnaire was translated into languages spoken by individual waste pickers. Participants’ consent was sought and an information sheet was provided to each waste picker by trained fieldworkers, who explained the study objectives and processes involved. Data was inputed directly into RedCap using an electronic structured questionnaire. RedCap is an electronic database capture and data management tool. The outcome variable, SRH question was used for participants to rate their health based on their experience in the previous 12 months. SRH was categorised as poor, fair, good and very good. The main explanatory variables were history of common infectious and chronic diseases and mental health categorised as yes or no. The infectious diseases included in the questionnaire were gastrointestinal and respiratory ailments, while diabetes, cancer, hypertension, stroke and asthma were the chronic diseases that were included. Individuals who reported at least one of the diseases were categorised as “yes” to history of infectious and chronic diseases. Twenty mental health questions were combined to form a score that screens for common mental illnesses. A positive result occurred if a participant responded yes to at least 8 of the questions based on World Health Organization Self-Reported Questionnaire tool for mental health [27]. Medical care access was defined as clinic visits in the previous 12 months.

Possible confounding factors collected were demographic factors (sex, age, and education), average monthly income in South African rand and then converted to United States (US) dollars, and years of work experience. Other variables included were ever smoking, alcohol consumption, eating food by the landfill site, landfill safety, cuts or injuries and PPE. The PPE questionnaire assessed whether the participants were using masks, gloves, and boots or closed shoes or nothing at all during waste picking. These variables were defined in a yes/no format as participants were asked about their past experience.

### 2.4. Statistical Analysis

Data cleaning and analysis were undertaken using Stata SE version 15.1(4905 Lakeway Drive, College Station, TX, USA). Descriptive statistics for continuous covariates were summarised as median and interquartile ranges (IQR) while categorical variables were described in number percentages. Inferential analyses were undertaken using ordinal logistic regression against the outcome variable and robust estimation of the standard errors to account for clustering in the two landfill sites. A model-building strategy was established using maximum likelihood ratio tests to reach a final multivariable model. Since age and years of work experience could be correlated, a pairwise correlation test was undertaken. Parallel multivariable analyses were undertaken with age and years of work separately to rule out the two variables linearly predicting each other. Effect estimates were reported as odds ratios (OR), and type 1 error (alpha) was considered at the 5% level of significance. Model goodness of fit test was determined. Difference between ‘clinic visits (yes/no) in the past 12 months’ among participants who reported an illness and at the same time rated their health as poor, was established.

## 3. Results

Table 1 shows a description of demographic, lifestyles and health outcomes among waste pickers. Of the 361 waste-pickers included in this study, 265 (73.41%) were males. The median age was 31 years (IQR: 27–39). The majority, 286 (79.44%), of the participants reached at minimum, secondary school education. The median income earned monthly was US $107 (IQR: 57–143). The median years of work at the landfill sites was 5 years (IQR: 3–10 years). The majority of the participants (86%) reported working at the landfill sites as unsafe. A higher proportion of the workers, 297 (82.73%) had experienced some injuries or cuts during work. A large percentage of the waste pickers (94.18%) used personal protective equipment during work. A total of 252 (69.81%) waste pickers reported having ever smoked and 116 (41.73%) drank alcohol. In the previous 12 months, just over half of the respondents sought medical care by visiting a clinic 208 (58.27%). A total of 132 (36.57%) workers were at risk of mental health problems. Chronic diseases were reported in 93 (25.56%) of the participants, while majority 303 (83.93%) reported to have suffered from infectious diseases. A percentage of 30% (107) of respondents perceived their health to be poor, fair 57 (15.92%), good 155 (43.30%), and very good 39 (10.89%).

Univariable analyses showed that SRH was significantly associated with landfill site safety, smoking, mental health, and chronic and infectious diseases (Table 2). Risk factors that were statistically significantly associated with SRH in the multivariable model among waste pickers are indicated in Table 2. Compared to non-smokers, waste pickers who smoked had 72% higher odds of reporting poor health with statistically significant differences adjusted odds ratio (AOR): 1.72; 95% confidence interval (CI): 1.11–2.66). The odds of reporting poor health were more than 40% lower among waste pickers who reported ever having cuts or injuries compared to those without any injuries (AOR: 0.53; 95% CI: 0.30–0.91). Individuals at risk of mental ill-health were 1.87 times more likely to report poor health compared to those not at risk of mental ill-health (AOR: 1.87; 95% CI: 1.22–2.84). Waste pickers who reported having suffered from chronic diseases had more than double the odds of reporting poor health unlike those who did not report chronic diseases (AOR: 2.34; 95% CI:1.47–3.68). Suffering from infectious diseases was statistically significantly associated with reporting poor health among these waste pickers (AOR: 2.07; 95% CI: 1.17–3.63). From a total of 316 who reported having experienced at least one illness, 99 (31%) of them rated their health as poor. From the 99, 40 (40.04%) visited a clinic in the previous 12 months. A comparison of the proportion between those who visited the clinic and those who did not showed marginally statistically significant differences (*p* = 0.0606).

## 4. Discussion

The objectives of this study were to investigate the association between illness, clinic visits and SRH, and to assess if poor self-rating of health increased clinic visits in the previous 12 months among waste pickers. Our study found that less than 30% of participants rated their health as poor. This finding is similar to that of a previous study which found that 34% rated their health as poor or fair [26]. However, another study reported that more than 50% of the waste pickers rated their health as poor [28]. It could be that in Johannesburg waste pickers do not perceive working at the landfill sites as a health hazard since the need for money takes precedence over their health. We found that 15% of the waste pickers rated their health as fair, contrary to a study by Mathema et al. [29] which reported that majority of the waste pickers perceived their health as fair. The study by Mathema et al. [29] further indicated that waste pickers were unable to report their health as poor due to possible fear of eviction from work, which could be another influential factor among the waste pickers in Johannesburg.

Despite the high burden of illnesses, the proportion of waste pickers who visited the clinic in the previous 12 months was low at 41% in Johannesburg. This finding is consistent with those conducted in Pretoria (41%) [29] and Colombia (32%) [30] where participants reported visiting a clinic or a hospital in the previous 12 months. However, a high proportion of clinic visits (63%) were observed in a Brazilian study [31]. These disparities might be attributed to the lack of health promotion and economic exclusion. These factors might have contributed to poor health care access among waste pickers in South Africa and elsewhere. Informal workers may find it time-wasting to take time off from work for medical consultation, since they are living on daily payments, where the principles of ‘no work, no money’ applies. Thus 40% of those who rated their heath as poor and reported a history of illnesses visited the clinic in the previous 12 months in our study.

Visiting a clinic or doctor during any ill-health episode can be one of the most important determinants of one’s health. In our study, no association between clinic visits and SRH was observed, which is similar to a previous study by Mahmoud. [32]. This is not surprising as waste pickers who reported injuries or cuts had decreased odds of reporting poor health by 47%. Ideally, injuries should have correlated with poor SRH and, therefore, show an association with increased odds of clinic visits. As reported elsewhere, waste pickers have a higher chance of being injured and contracting other occupational-related illnesses compared to individuals in other sectors [33]. Injuries in our study resulted from needles and sharp objectives including broken glasses and many of the waste pickers reported using traditional herbs in self-treatment to heal injuries. Ineffective treatment can result in bacterial infections which exacerbates the poor health conditions. However, waste pickers might have coping strategies towards injuries and other common diseases which comes with years of work experience in the landfill sites and use of traditional or self-medication [34].

Reported history of common infectious diseases had increased odds of poor SRH compared to those who did not report any infectious diseases. A longitudinal study also reported that individuals with poor SRH were 3 times more likely to develop infectious diseases [35]. Direct contact with dangerous waste, in particular medical waste at the landfill sites, might increase the risk of infection [36]. Contamination by heavy metals and dangerous waste put waste pickers at risk of developing pulmonary diseases, human immunodeficiency virus (HIV) and hepatitis C as a result of contact with sharp items and hazardous health waste [16], and is made even worse by inadequate PPE which leads to injuries or cuts [37]. Waste pickers in South Africa mostly collect broken or old shoes and clothes from the landfill sites and use these as PPE which are not effective for protection against injuries, cuts and exposure to infections. Gastrointestinal diseases and respiratory infections are the major aliments reported in this study. Respiratory infections are one of the most common infections among waste pickers and they might be predisposed to this through burning waste and inhaling the smoke [30]. In addition, infectious diseases might arise from waste pickers eating recovered food from the landfill sites, increasing the risk of gastrointestinal problems such as diarrhoea [38]. One study found that the incidence of diarrhoea is 10 times greater among waste pickers than in the general population [33].

Participants with a history of chronic diseases were at least 2 times more likely to report poor health than with those no history of chronic diseases. Limited research has focused on the association between chronic diseases and SRH in the waste pickers. Machado et al. [39] reported an association between chronic diseases and poor SRH. A study from India also reported an association between SRH and chronic disease [40]. Chronic diseases like diabetes and hypertension were significantly associated with reporting poor health and were the most prevalent among waste pickers [12,41]. Landfill sites contain chemicals which might have adverse effects on the environment. They pollute the air by emitting gases like methane. This methane is a result of the burning of waste, and this in turn results in asthma, cancer, chronic coughs and other respiratory diseases [29,42,43].

Waste pickers who were at risk of mental ill health were more likely to report poor personal health status. This is in agreement with a previous study from India which found a relationship between mental health and SRH [40]. Waste pickers in Guatemala reported fewer visits to clinics due to perceived discrimination by clinic staff which could cause stress and depression [19]. A study reported that psychiatric problems like depression and anxiety are common among waste pickers, which are caused by stigmatization [44]. Furthermore, other studies found a significant relationship between mental ill health like depression, and perceived health status in the general population [45].

The strength of this study was that a structured and validated questionnaire was used to collect data. This would make our findings valid and comparable to other studies that used similar tools. One of the limitations is that the results may not be generalizable enough because we only sampled participants from two landfill sites. In addition, there could also be information bias as waste pickers may not be truthful in responding during the data collection because of fear of any potential repercussion from the landfill management. Also the prevalence of diseases might have been underestimated because of recall bias resulting from a self-reported history of diseases. In addition, the self-reported nature might have resulted in a social desirability bias where some participants might have over-reported their history of clinic visits so they would be viewed as good by the research interviewers.

## 5. Conclusions

Reported history of acute and chronic illnesses including mental health were associated with poor SRH but this did not increase clinic visits in the previous 12 months. Provision of mobile clinic services at the landfill sites could increase access to medical health care for the waste pickers.

## Figures and Tables

**Table 1:** Description of demographic, lifestyle and health outcomes.

Characteristics	Total (*n* = 361)
Sex	
Male	265 (73.41%)
Female	96 (26.59%)
Age (median (IQR) years)	31 (27–39)
Education	
None	15 (4.17%)
Primary	59 (16.39%)
Secondary	286 (79.44%)
Income (median (IQR) *)	107 (57–143) *
Years of work (median (IQR))	5 (3–10)
Landfill site eating	
No	137 (38.70%)
Yes	217 (61.30%)
Landfill safety	
No	303 (85.59%)
Yes	51 (14.41%)
Personal protective equipment	
No	21 (5.82%)
Yes	340 (94.18%)
Ever smoked	
No	109 (30.19%)
Yes	252 (69.81%)
Alcohol use	
No	162 (58.27%)
Yes	116 (41.73%)
Cuts or injuries	
No	62 (17.27%)
Yes	297 (82.73%)
Clinic visits in the previous 12 months	
No	208 (58.76%)
Yes	146 (41.24%)
Mental health	
Not at risk	229 (63.43%)
At risk	132 (36.57%)
Chronic diseases	
No	268 (74.24%)
Yes	93 (25.56%)
Infectious diseases	
No	58 (16.07%)
Yes	303 (83.93%)
Self-rated health	
Very good	39 (10.89%)
Good	155 (43.30%)
Fair	57 (15.92%)
Poor	107 (29.89%)

IQR: Interquartile ranges; * United States dollar ($).

**Table 2:** Investigation of the association between illness, clinic visits and self-rated health.

Risk Factors	Univariable Analyses	Multivariable Final Analyses
	OR (95% CI)	AOR (95% CI)
Sex		
Male	1 (ref)	
Female	0.92 (0.59; 1.41)	
Age/10	0.95 (0.78; 1.15)	
Education		
None	0.57 (0.21; 1.52)	
Primary	0.79 (0.46; 1.33)	
Secondary	1 (ref)	
Income	1.00 (0.86; 1.17)	
Years of work	0.98 (0.95; 1.02)	
Landfill site eating		
No	1 (ref)	
Yes	1.39 (0.93; 2.07)	
Landfill safety		
No	1 (ref)	
Yes	0.56 (0.10; 0.97) *	
Cuts or injuries		
No	1.23 (0.73; 2.04)	
Yes	1 (ref)	
PPE		
No	1.31 (0.51; 3.34)	
Yes	1 (ref)	
Ever smoked		
No	1 (ref)	1 (ref)
Yes	1.57 (1.03; 2.39) *	1.72 (1.11; 2.66) *
Alcohol use		
No	1 (ref)	
Yes	1.00 (0.65; 1.55)	
Injuries or cuts		
No	1 (ref)	1 (ref)
Yes	0.81 (0.48; 1.35)	0.53 (0.30; 0.91) *
Clinic visits in the previous 12 months		
No	1 (ref)	
Yes	1.13 (0.76; 1.66)	
Mental health		
Not at risk	1 (ref)	1 (ref)
At risk	2.02 (1.35; 3.01) **	1.87 (1.22; 2.84) **
Chronic diseases		
No	1 (ref)	1 (ref)
Yes	2.38 (1.53; 3.69) ***	2.34 (1.47; 3.68) ***
Infectious diseases		
No	1 (ref)	1 (ref)
Yes	2.41 (1.39; 4.16) **	2.07 (1.17; 3.63) *

* *p* < 0.05; ** *p* ≥ 0.0001; *** *p* < 0.0001, OR: Odds ratio, AOR: Adjusted odds ratio, CI: Confidence intervals; PPE: Personal protective equipment; Correlation between age and years of work (β = 0.5590; *p* < 0.0001); Parallel regression assumption (*p* = 0.890); ref: reference.
